# Common mental disorders among medical students in Jimma University, SouthWest Ethiopia

**DOI:** 10.4314/ahs.v17i3.27

**Published:** 2017-09

**Authors:** Habtamu Kerebih, Mohammed Ajaeb, Hailemariam Hailesilassie

**Affiliations:** 1 Department of Psychiatry, College of Health Science, Jimma University, Ethiopia; 2 Laska Meles Zenawi memorial primary Hospital, South Nation Nationalities And People Region, South Ethiopia

**Keywords:** Common mental disorders, medical students, prevalence, Ethiopia

## Abstract

**Background:**

Medical students are at risk of common mental disorders due to difficulties of adjustment to the medical school environment, exposure to death and human suffering. However there is limited data on this aspect. Therefore, the current study assessed the magnitude of common mental disorders and contributing factors among medical students.

**Methods:**

An institutional based cross-sectional study was conducted from May 12–16, 2015 using stratified sampling technique. Three hundred and five medical students participated in the study. Common mental disorders were assessed using the self-reported questionnaire (SRQ-20). Logistic regression analysis was used to identify factors associated with common mental disorders among students. Adjusted odds ratios with 95% confidence interval were computed to determine the level of significance.

**Result:**

Prevalence of common mental disorders among medical students was 35.2%. Being female, younger age, married, having less than 250 birr monthly pocket money, attending pre-clinical class, khat chewing, smoking cigarettes, alcohol drinking and ganja/shisha use were significantly associated with common mental disorders.

**Conclusion:**

The overall prevalence of common mental disorders among medical students was high. Therefore, it is essential to institute effective intervention strategies giving emphasis to contributing factors to common mental disorders.

## Background

Common mental disorders (CMD) include anxiety, depression, and somatic symptoms, including sleep problems, headache, and backache^1^–^3^; which are the leading cause of disability worldwide^4^. There is evidence that mental disorders can lead to increased morbidity and mortality, especially the risk of death by suicide in persons with depression, anxiety or substance abuse. Various study results revealed that students of higher institutions pass through a number of difficulties which may lead to drop out from the college or overt mental illnesses^5^. Furthermore, factors such as leaving guardians start to live in dormitory setting, academic load, constant pressures to succeed, competition with peers, financial burden, peer, teacher or parental pressure as well as concern about the future were found to significantly contribute to psychological or mental distress^6^,^7^. Medical students are prone to CMD due to adjustment to the medical school environment, ethical conflict, and exposure to death, human suffering and problems in life. These could lead to impaired academic performance, substance abuse, academic dishonesty and even suicide^8^.

Available studies conducted to assess CMD indicated that the prevalence of CMD among medical students is high. A study conducted in Brazil among final year health care students which included dentistry, nursing and medical students showed that the prevalence of CMD was 33.7%^9^. Even though, significant a relatively low prevalence (14.5–15.04%) rate of CMD was reported from a study done in India^10^,^13^. However, other studies conducted among medical students such as study results from Iran (49.5%) and Karnataka (32.2%) consistently indicated higher prevalence of CMD^11^,^12^. A prevalence rate of 29.3% of CMD was also reported among Universidad Federal da Bahia medical students^14^.

In Ethiopia, there are prevalence studies of CMD done among undergraduate university students. The study conducted in University of Gondar, NorthWest Ethiopia showed 40.5% prevalence of CMD and another study conducted in Adama University, Eastern Ethiopia indicated 21.6% of CMD among undergraduate students^15^,^16^. A study conducted among Addis Ababa University medical students in 2001 reported that 32.6% of the students had mental distress^5^.

Study results revealed that coming from rural areas^10^, having financial distress, family history of mental illness, ever khat use, ever shisha use^15^,^16^, being female, being a preclinical student and being less than 20 years of age^5^ were found to significantly contribute to CMD.

Studies about common mental disorders among Ethiopian medical students, who are at risk because of academic overload and dealing with human suffering, are very limited and were conducted 15 years ago only in Addis Ababa university^5^. Currently, in Ethiopia, the number of students joining medical school is growing more than ever. For appropriate intervention to take place there should be epidemiological studies which show the burden of CMD among medical students over time and in different settings or universities in the country. Therefore, the current study assessed the prevalence of common mental disorders and associated factors among medical students of Jimma University which can be used as an input for policy makers and stakeholders working in health and higher education programs.

## Methods

### Study design and setting

An institutional-based cross sectional study design was conducted among medical students in Jimma University from May 12 to 16 of 2015. Jimma University is located in Jimma town which is 352 km from Addis Ababa, the capital city of Ethiopia. The medical school at Jimma University (formerly Jimma Institute of Health Sciences) was launched in 1983. The university is one of the most reputable centers of excellence in medical education in the country. In the study period a total of 1544 medical students were registered in the college from first year to year six.

### Participant selection

All medical students (from first year to internship) registered in the year 2014/2015 were the source population. Medical students included into the sample and from whom data was obtained were the study population. Based on their training background, students were divided into two groups: pre-clinical (Year I and II) and clinical (Year III, Year IV and internship).

### Sampling

Taking prevalence rate of 32.6% for common mental disorders from a study done among Addis Ababa university medical students^5^, at 95% certainty and ±5% margin of error and using the population correction formula and by adding 10% non-response rate the final sample size was 305. Six strata were formed based on year of study of medical students. Then medical students were proportionally selected from each year of study using simple random sampling technique.

### Data collection tools

The Amharic version of Self-Reporting Questionnaire (SRQ) with 20 items was used to assess the level of common mental disorders among medical students. The SRQ is a 24 item questionnaire developed by the World Health Organization to screen for emotional distress^17^. Originally, SRQ was constructed to screen psychotic (4 items) and non-psychotic (20 items) disorders. Because of the low sensitivity and unreliability of the 4 psychotic items, the 20 items are widely used in epidemiologic studies, hence referred as SRQ-20. The SRQ-20 was translated and validated for use in Ethiopia^18^. The SRQ-20 asks respondents whether, within the 4 weeks before the data collection date, they had experienced symptoms associated with emotional distress, such as crying, inability to enjoy life, tiredness, and suicidal thoughts. A cut-off point of 7/8 (7‘yes's’ a non-case, 8 ‘yes's’ a case) was used which is the most commonly used cut off point in developing countries^19^. This cut off point was also used in previous studies in Ethiopian Universities^15^,^20^.

### Data collection procedures

Before the actual data collection took place, the questionnaire was pre-tested on 5% of Dentistry students to check for applicability and understandability of the assessment tools. During the actual data collection, questionnaire was provided to randomly selected participants by trained data collectors. Once the students filled the self administered questionnaire, the data collectors checked if there was incomplete information and missed values on spot for which they returned to the participant to re fill the missed values

### Statistical analysis

Upon completion of data collection, data was coded and entered into statistical package for social sciences (SPSS version 20). Then, data was analyzed to generate descriptive statistics such as mean, frequencies and percentages. To determine the association between dependent and independent variables, variables were entered into the model one by one in simple logistic regression analysis. Then variables with p-value <0.25 in simple logistic regression analysis were entered into multiple logistic regression analysis using enter method. Finally, variables with p-value <0.05 in multivariate regression analysis were declared to have statistically significant association with the outcome variable. The strength of association was measured using odds ratios at 95% confidence interval.

### Ethical approval

The ethical approval was obtained from the research ethical review board of the College of Health Sciences of Jimma University. An information sheet was attached with each questionnaire to provide the study details and rights of the study participants. Written informed consent was obtained from the study participants. Data was kept anonymous and confidential during all stages of the study.

## Results

### Background characteristics of study participants

Data were obtained from 290 medical students with a response rate of 95%. The age of the participants ranged from 18 to 30 years and 71.7% them were within 18 to 24 years. The majority of the students were male (70.7%), Oromo (40.7%) and single (94.8%). More than half 59.7% and 60.7% of the students were pre-clinical and originally from urban areas, respectively (Table 1).

**Table 1:** Socio demographic characteristics and substance use among medical students of Jimma University (n=290), May, 2015.

Variables		Frequency	Percentage
**Sex**	Male Female	205 85	70.7 29.3
**Age**	18–24 >24	208 192	71.7 28.3
**Religion**	Orthodox Protestants Catholic Muslim Others*	120 64 7 92 7	41.4 22.1 2.4 31.7 2.4
**Ethnicity**	Oromo Amhara Gurage Tigre Wolayta Somale Others **	118 76 45 13 14 11 13	40.7 26.2 15.5 4.5 4.8 3.8 4.5
**Marital status**	Single Married	275 15	94.8 5.2
**Year of study**	Pre-clinical Clinical	173 117	59.7 40.3
**Place of origin**	Urban Rural	176 114	60.7 39.3
**Income(monthly** **pocket money)**	<250 250–500 501–750 751–1000 >1000	27 122 73 27 41	9.3 42.1 25.2 9.3 14.1
**Family history of** **mental illness**	Yes No	11 279	3.8 96.2
**Chat chewing**	Yes No	26 264	9.0 91.0
**Cigarette smoking**	Yes No	25 265	8.6 91.4
**Alcohol**	Yes No	39 251	13.4 86.6
**Shisha/ganga**	Yes No	19 271	6.6 93.4

Other*= Wakefata and Pagan, other**= Yem, Hadiya and Dawuro,

### Prevalence of common mental disorders and associated factors among medical students

The prevalence of common mental disorders was 35.2%. Out of the study samples, 102 students reported mental distress, using the aforementioned SRQ-20 cut off points. A large proportion of young (42.3%), female (60.0%) and pre-clinical (45.1%) medical students reported CMD than their counter parts. In addition, a large number (59.3% of students with < 250 Ethiopian birr monthly pocket money and those students who were originally from rural areas (47.7%) had higher CMD. On the other hand, larger proportion of medical students with a family history of mental illness (72.7%) had higher rate of CMD than students without a history of mental illness in the family. In this study, as compared to non-substance users, larger proportion of medical students who had chewed khat (69.2%), smoked cigarette (80%), drank alcohol (69.2%), and used ganja/shisha (84.2%) were found to have a higher rate of CMD. (Table 2).

**Table 2:** Prevalence of common mental disorders associated with socio-demographic characteristics and substance use (n=290), May, 2015

Variable	Category	Mental distress	
		Yes	No
Age	18–24 >24	88 (42.3%) 14(17.1%)	120(57.7%) 68(82.9%)
Sex	Male Female	51(24.9%) 51(60.0%)	154(75.1%) 34(40.0%)
Marital status	Single Married	91(33.1%) 11 (73.3%)	184(66.9%) 4 (26.7%)
Religion	Muslim Orthodox Protestants Catholic Others*	22(23.9%) 48(40.0%) 29(45.3%) 2(28.6%) 1(14.3%)	70(76.1%) 72(60.0%) 35(54.7%) 5(71.4%) 6(85.7%)
Ethnicity	Oromo Amhara Tigre Somale Wolayta Gurage Others	45(38.1%) 29(38.2%) 4(30.8%) 10(90.9%) 4(28.6%) 16(35.6%) 3(23.1%)	73(61.9%) 47(61.8%) 9(69.2%) 1(9.1%) 10(71.4%) 29(64.4%) 10(76.9%)
Year of study	Pre-clinical Clinical	78(45.1%) 24(20.5%)	95(54.9%) 93(79.5%)
Pace of origin	Urban Rural	48 (273%) 54 (47.4%)	128(72.7%) 60(52.6%)
Family history of mental illness	Yes No	8(72.7%) 94(33.7%)	3(27.3%) 185(66.3%)
Income (monthly pocket money)	<250 251–500 501–750 751–1000 >1000	16 (59.3%) 38 (31.1%) 29 (39.7%) 5 (18.5%) 14 (34.1%)	11(40.7%) 84(68.9%) 44(60.3%) 22(81.5%) 27(65.9%)
Khat chewing	Yes No	18(69.2%) 84 (31.8%)	8(30.8%) 180(68.2%)
Cigarette smoking	Yes No	20 (80.0%) 82 (30.9%)	5(20.0%) 183(69.1%)
Alcohol drinking	Yes No	27 (69.2%) 75 (29.9%)	12(30.8%) 176(70.1%)
Shisha/ ganga use	Yes No	16 (84.2%) 86 (31.7%)	3(15.8%) 185(68.3%)

### Predictors of common mental disorders among medical students

Logistic regression analysis of outcome variable against predictors indicated that the probability of having CMD was about 4 times more likely among female medical students than males (AOR=3.94, 95% CI: 1.92–8.12). Younger medical students were also 3.3 times more vulnerable to developing CMD than their counter parts (AOR=3.32, 95%CI: 1.40– 7.91). The probability of CMD among married students was 5 times (AOR=5.17, 95%CI: 1.09–24.50) higher than singles. Besides, students in the pre-clinical classes were 3 times more prone to having CMD than clinical class students (AOR=3.39, 95%CI: 1.53–7.51). Students with monthly pocket money of less than 250 Ethiopian Birr were 5.6 times more vulnerable to CMD (AOR=5.62, 95%CI: 1.24–25.47) than students with higher monthly pocket money. On the other hand, medical students who chewed khat were 7 times more likely to have CMD than non-khat users (AOR=6.91, 95%CI=1.88– 25.42). The odds of having CMD was 9 times greater among medical students who smoke cigarette than non-smokers (AOR=8.93, 95%CI: 3.24- 24.61). Besides, medical students who drink alcohol had a 5 times risk of developing CMD (AOR=5.28, 95% CI: 2.54–10.98) than non-drinkers. Whereas, ganja/shisha users were about 11 times (AOR=11.47, 95%CI: 3.26–40.42) more vulnerable to CMD than their counter parts (Table 3)

**Table 3:** Logistic regression analysis on factors predicting common mental disorders among medical (n=290), May, 2015

Variable	Category	COR(95%CI)	P-value	AOR (95%)	P-value
Sex	Male Female	1.00 4.53(2.65–7.75)	<0.001*	3.94 (1.92–8.12)	<0.001*
Age	18–24 >24	3.56(1.88–6.74) 1.00	<0.001*	3.32 (1.40–7.91)	0.007*
Marital status	Single Married	1.00 0.18(0.06–0.58)	0.004*	5.17 (1.09–24.50)	0.039*
Religion	Muslim Orthodox Protestants Catholic Others	1.89(0.22–16.52) 4.00(0.47–34.28) 4.97(0.57–43.69) 2.400(0.16–34.93) 1.00	0.567 0.206 0.148 0.522	1.04 (0.09–11.7) 2.75 (0.24–31.70) 1.68 (0.14–20.17) 0.93 (0.04–23.06)	0.972 0.418 0.683 0.966
Ethnicity	Oromo Amhara Tigre Somale Wolayta Gurage Others	6.16(0.76–49.79) 6.17(0.75–50.75) 4.44(0.42–47.50) 1.00 4.00(0.38–42.37) 5.52(0.65–47.10) 3.00(0.27–33.97)	0.088 0.091 0.217 0.250 0.119 0.375	8.30 (0.67–102.95) 6.42(0.50–81.67) 4.02(0.23–69.87) 16.40(0.94–286.73) 6.58(0.49–88.85) 6.63(0.34–128.75)	0.099 0.152 0.339 0.055 0.156 0.211
Year of study	Pre-clinical Clinical	3.18(1.86–5.46) 1.00	<0.001*	3.39(1.53–7.51)	0.003*
Place of origin	Urban Rural	1.00 2.40(1.46–3.94)	0.001*	1.59(0.79–3.19)	0.193
Income (monthly Pocket money)	<250 251–500 501–750 751–1000 >1000	6.40(1.86–22.10) 1.99(0.70–5.65) 2.90(0.99–8.53) 1.00 2.28(0.71–7.32)	0.003 0.196 0.053 0.166	5.62 (1.24–25.47) 1.39 (0.40–4.82) 2.05 (0.53–7.86) 4.41 (0.98–19.93)	0.025* 0.603 0.296 0.054
Family history of mental illness	Yes No	5.25(1.36–20.24) 1.00	0.016	4.03 (0.58–27.80)	0.158
Khat chewing	Yes No	4.82(2.02–11.53) 1.00	<0.001*	6.91 (1.88–25.42)	0.004*
Cigarette smoking	Yes No	8.93(3.24–24.61) 1.00	<0.001*	4.38 (1.20–16.01)	0.026*
Alcohol drinking	Yes No	5.28(2.54–10.98) 1.00	<0.001*	5.79 (2.10–15.99)	0.001*
Shisha/ ganga use	Yes No	11.47(3.26–40.42) 1.00	<0.001*	5.02 (1.17–21.56)	0.030*

1.00= reference,

* p <0.05

## Discussion

In this study, the prevalence of common mental disorders (CMD) among medical students was 35.2% using SRQ-20 cut off point of 7/8. This is similar to previous study findings from Ethiopia, among medical students of Addis Ababa University in which the prevalence of CMD was 32.6%^5^ and among Gondar university undergraduate students (40.9%)^15^. It was also in agreement with study results reported from South America among Brazil health care students (33.7%) and medical students^14^. In a study conducted among medical students of a private college in South Karnataka, South Western region of India similar CMD prevalence rate (29.6%) was reported provided that the later used SRQ-20 cut off point of 9/10^11^.However, the prevalence of CMD in this study was higher than previous research findings done in Eastern Ethiopia, among Adama university undergraduate students (21.6%)^16^. The probable reason for the discrepancy could be that the current study focuses on medical students who deal with human health conditions in addition to academic work load while the previous study was on undergraduate students from all fields of study. The other reason could be the use of different cut off point for the SRQ-20 in which the previous study used a cut-off points of ≥ 11. The current CMD prevalence rate was also higher in studies done outside Ethiopia among medical students in Punjab India (15.04%)^10^ and in Kolkata India (14.5%)^13^. The reason could be that the two Indian studies used SRQ-20 cut off point of ≥ 10 while the current study used a cut-off point of ≥8. On the other hand, the current study finding is lower than previous study done among Iranian medical students (49.5%)^12^. This might be the use of different assessment tools in which Iranian study used the General Health Questionnaire-12 items (GHQ-12) in assessing prevalence of CMD.

Regarding factors affecting mental distress, being female was significantly associated with higher rates of CMD than being male. Various studies also reported that CMD was more frequent in females than in males^10^,^12^,^15^. Medical students within the age group of 18–24 years were more affected by CMD than students with > 24 years. This was similar to the result reported from the South Karnataka study^11^. Similarly, different study results revealed that CMD prevalence is higher in students who were in preclinical level of medical training particularly in first and second years^10^,^11^,^15^. The current study also showed that medical students with pre-clinical level of medical training were more affected by CMD than medical students with clinical level of medical training. Therefore, the probable reason for CMD being higher in younger medical students could be related to the level of medical training in which younger age groups of students are found in pre-clinical than clinical level of training where they are academically strained together with difficulty of environmental and social adaptability of campus life. Besides, being a married medical student significantly predicted a higher probability of developing CMD than singles. However, previous studies conducted among medical students in Iran and Brazil indicated that there were no differences in prevalence rate of CMD among married and single medical students^12^,^14^. The probable reasons for the difference could be the difference in socio-culture among the study populations. The other reason could be that most married students in the current study, where the beloved one is considered to be a strong source of social and emotional support in his/her physical presence, might be far away during the time of academic, social and environmental difficulties. In this study, the prevalence of CMD was higher in students who get less than 250 Ethiopian birr pocket money per month than students who get more. This is in line with a study from Nigeria where it was indicated that mental distress was higher in students who had financial distress^21^. The costs associated with stationery materials, photocopy service and students' sense of helplessness might be contributing factors for higher prevalence of CMD. Substance use, such as khat chewing, cigarette smoking, alcohol use and shisha/ganga use were found to increase the risk of CMD. Previous studies also showed similar findings particularly with khat chewing and CMD^15^,^16^.

However, the findings of this study must be interpreted taking into account the following limitations: recall bias due to the nature of the assessment tool which required recalling encounters in the past one month. Even though, self administered, it might have social desirability bias particularly related to substance use.

## Conclusion

Common mental disorders appear to be common among medical students. The distribution of CMD among medical students showed that it was higher in females, relatively younger age groups, pre-clinical level of medical training, married, low monthly pocket money and in students with substance abuse. Therefore, the mental health well being of medical students needs to be cautiously explored and due attention has to be given to design intervention programs aimed at addressing the identified risk factors.
